# Supplementary material for: A flat carborane with multiple aromaticity beyond Wade–Mingos’ rules
Source: Nat Commun. 2020 Jul 6;11:3370. doi: 10.1038/s41467-020-17166-9 (PMC7338440; doi:10.1038/s41467-020-17166-9)
Supplement: Supplementary file 1 — Supplementary Info [file 41467_2020_17166_MOESM1_ESM.pdf]

Supplementary Information for

A Flat Carborane with Multiple Aromaticity beyond Wade—Mingos' Rules

Lu et al.

This PDF file includes:

Supplementary Methods

Supplementary Figures

Supplementary Tables

Supplementary References

Supplementary Methods:

Crystallographic details X-ray data collection and structural refinement. Intensity data for compounds **2** and **3** were collected using a Bruker Kappa Apex II duo diffractometer. The structures were solved by Intrinsic Phasing (SHELXTL-2014) (*1*) and refined for all data by full-matrix least squares methods on F^2. (*2*) All non-hydrogen atoms were subjected to anisotropic refinement. The hydrogen atoms were generated geometrically and allowed to ride in their respective parent atoms; they were assigned appropriate isotropic thermal parameters and included in the structure-factor calculations.

Computational details

Gaussian 09 (Revision E.01) was used for all density functional theory (DFT) calculations. (*3*) Geometry optimizations and frequency calculations (with no restraints), natural bond orbital (NBO), and nucleus-independent chemical shifts (NICS) analysis of simplified model compounds **opt-2'**, **opt-2'(H)**, **3'**, **2''** and **III-V** were performed at the B3LYP/6-311G** level of theory, whereas the triplet ground state model compound **2'T** was optimized at the UB3LYP/6-311G** level of theory. For simplification of **opt-2'**, **3'** and **2'T**, the 2,6-diisopropyl phenyl groups of the BNC_3 five-membered rings of **2** and **3** were replaced with phenyl groups.

Supplementary Figures:

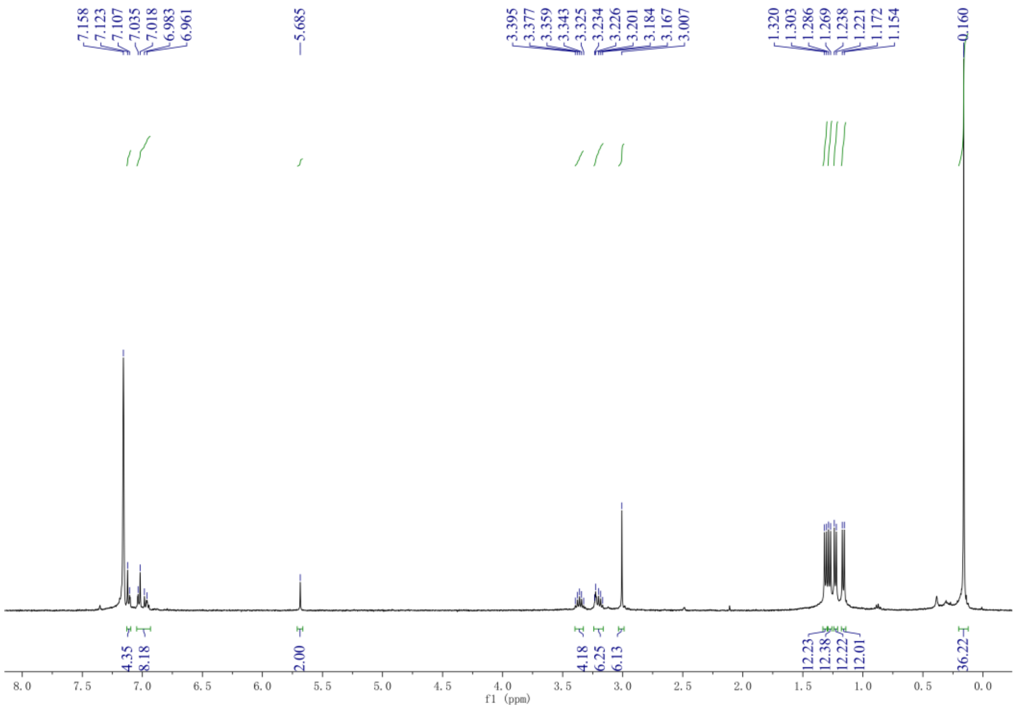

Supplementary Figure 1. ^{1}H NMR spectrum of **2** in C_6D_6

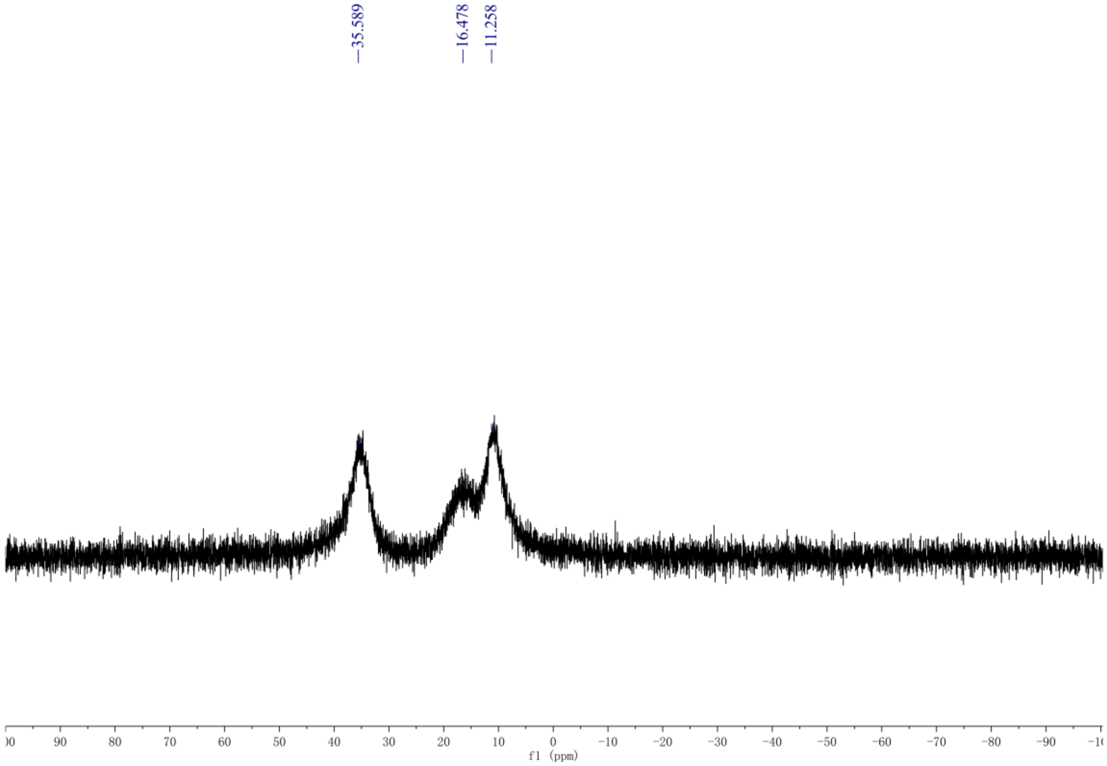

Supplementary Figure 2. ^{11}B NMR spectrum of **2** in C_6D_6

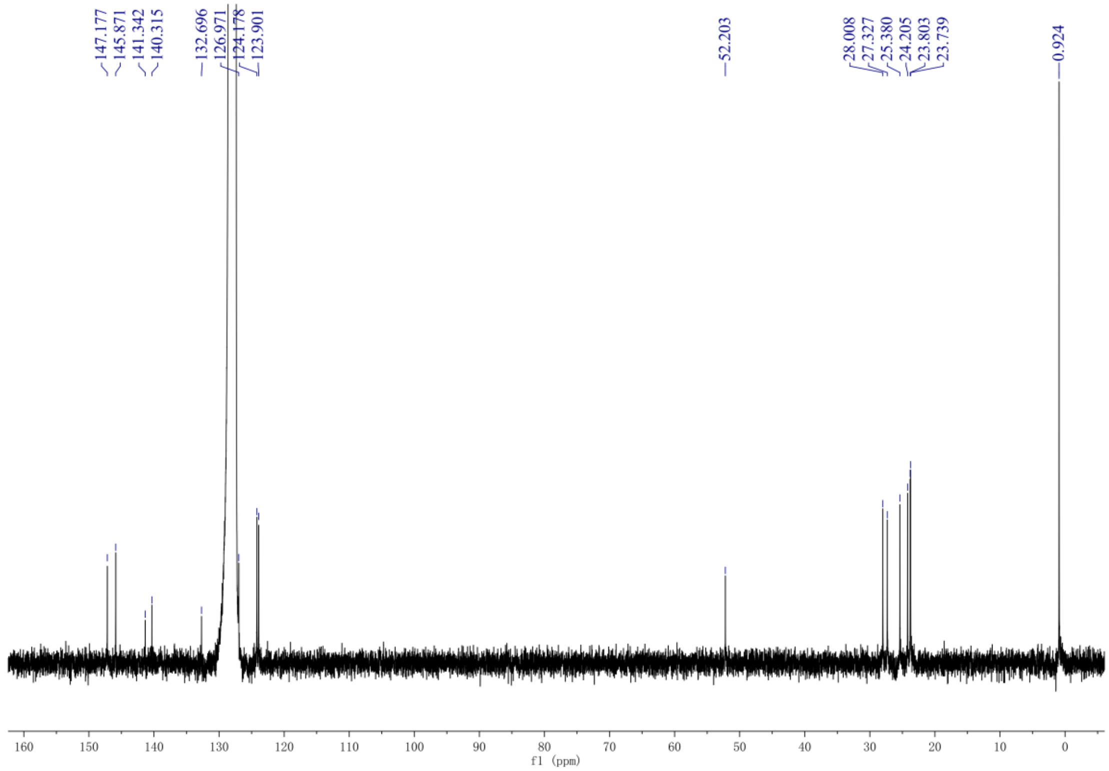

Supplementary Figure 3. $^{13}C\{^{1}H\}$ NMR spectrum of **2** in C_6D_6

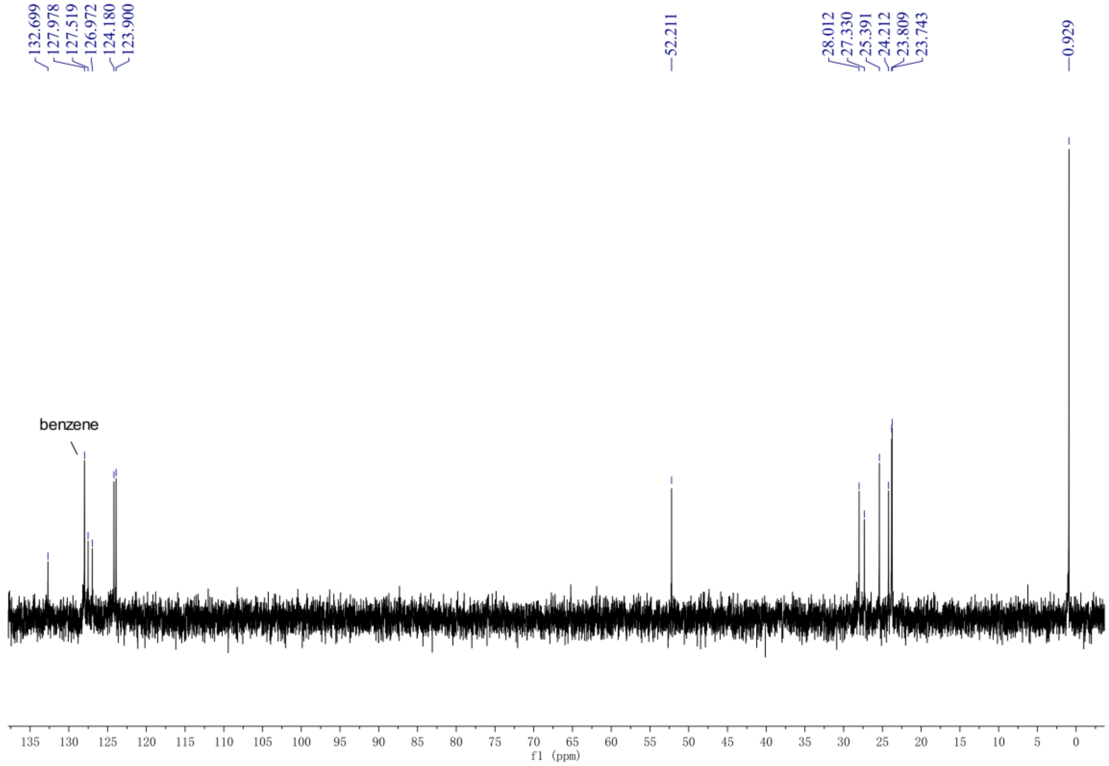

Supplementary Figure 4. $^{13}C\{^{1}H\}$ NMR (DEPT 135) spectrum of **2** in C_6D_6

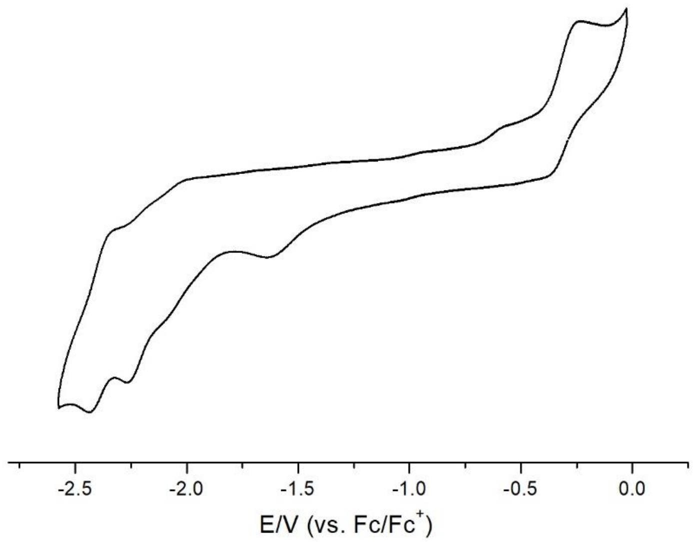

Supplementary Figure 5. Cyclic voltammogram of **2** in THF/0.1 M [$^{n}Bu_4N$][PF_6] at room temperature. Scan rate: 100 mVs^{-1}.

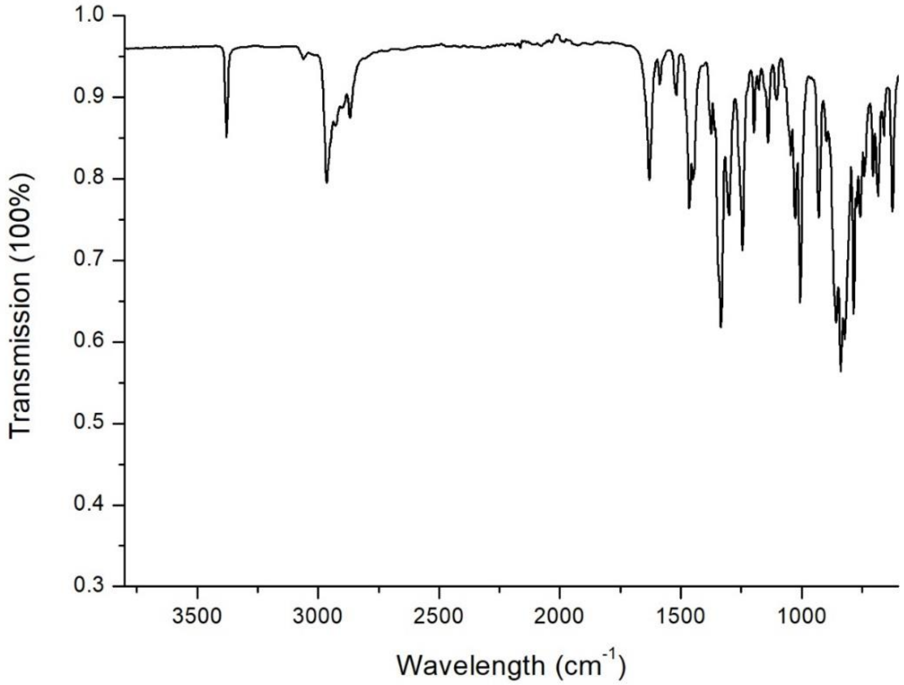

Supplementary Figure 6. FT-IR spectrum of **2**.

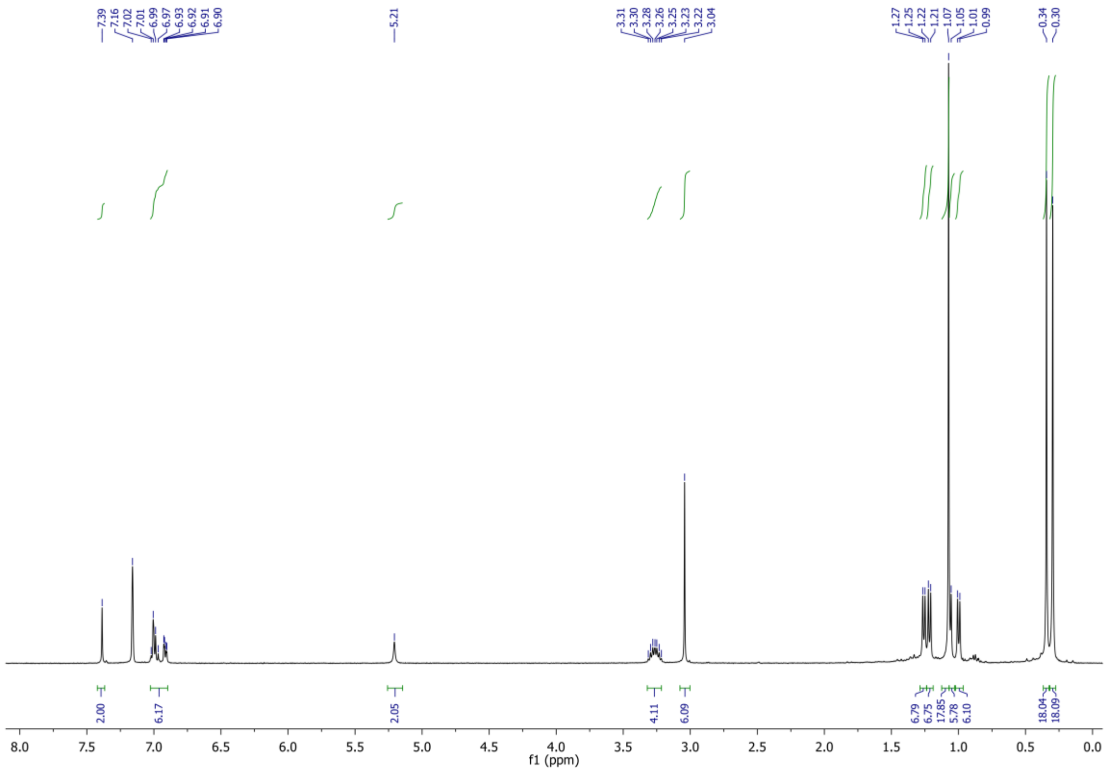

Supplementary Figure 7. ^{1}H NMR spectrum of **3** in C_6D_6

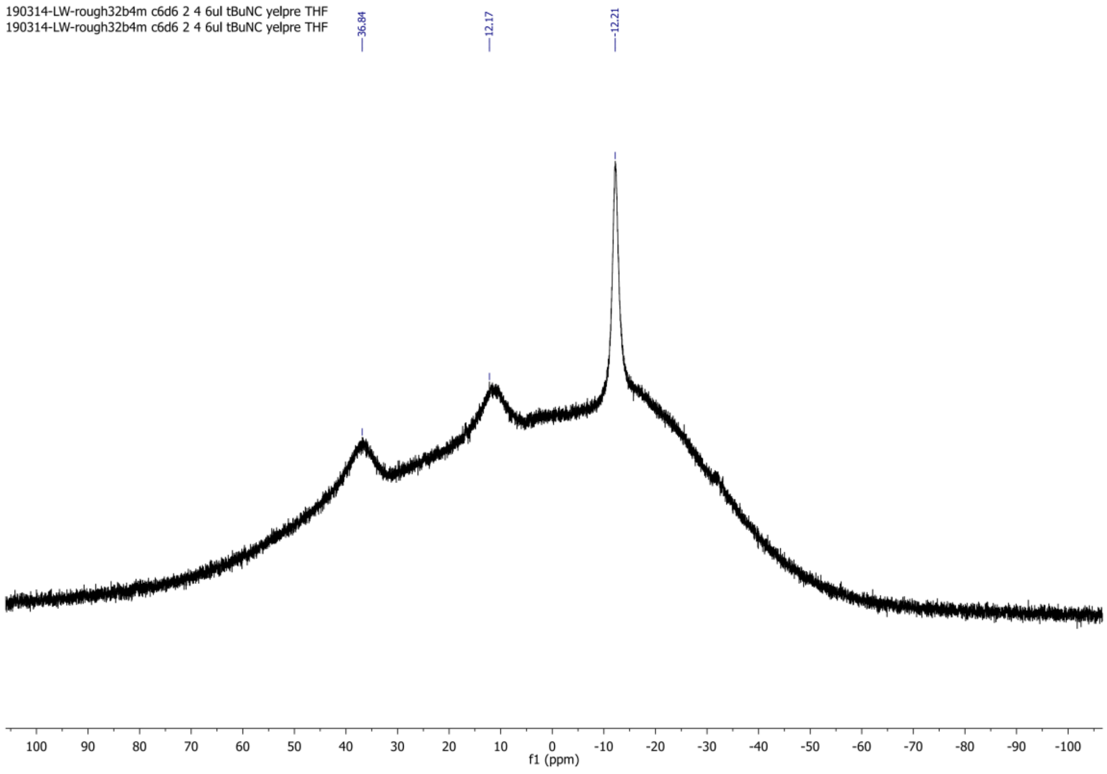

Supplementary Figure 8. ^{11}B NMR spectrum of **3** in THF-d_8

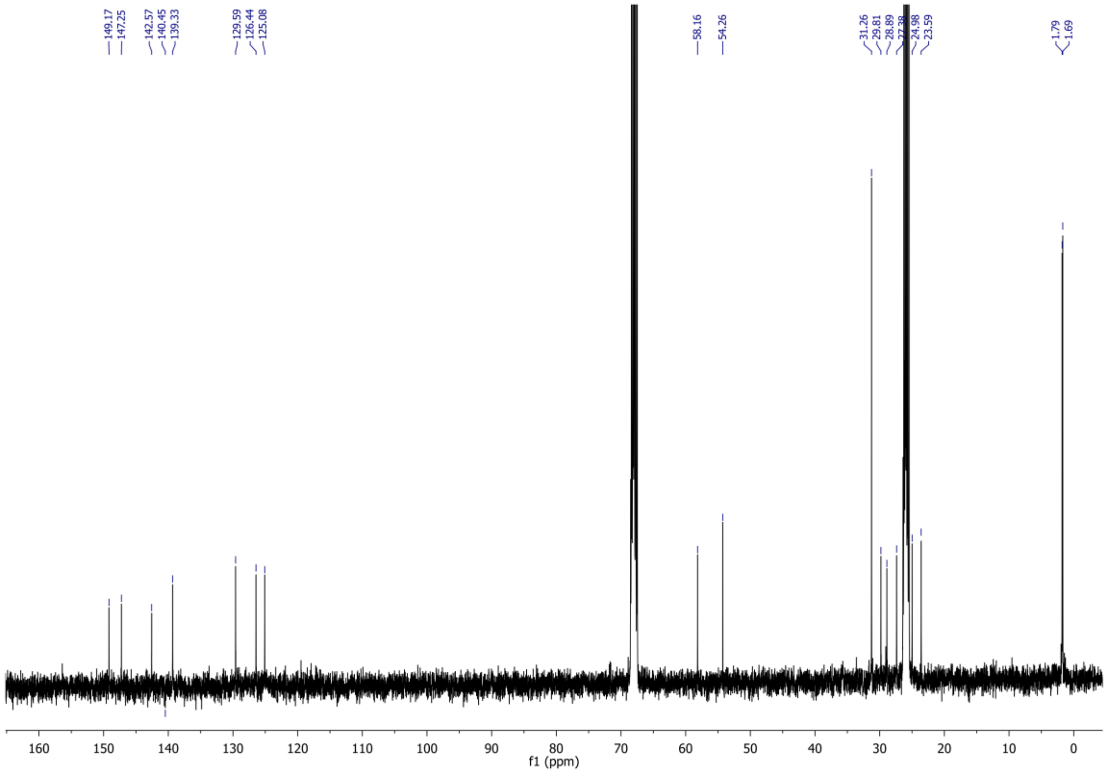

Supplementary Figure 9. $^{13}C\{^{1}H\}$ NMR spectrum of **3** in THF-d_8

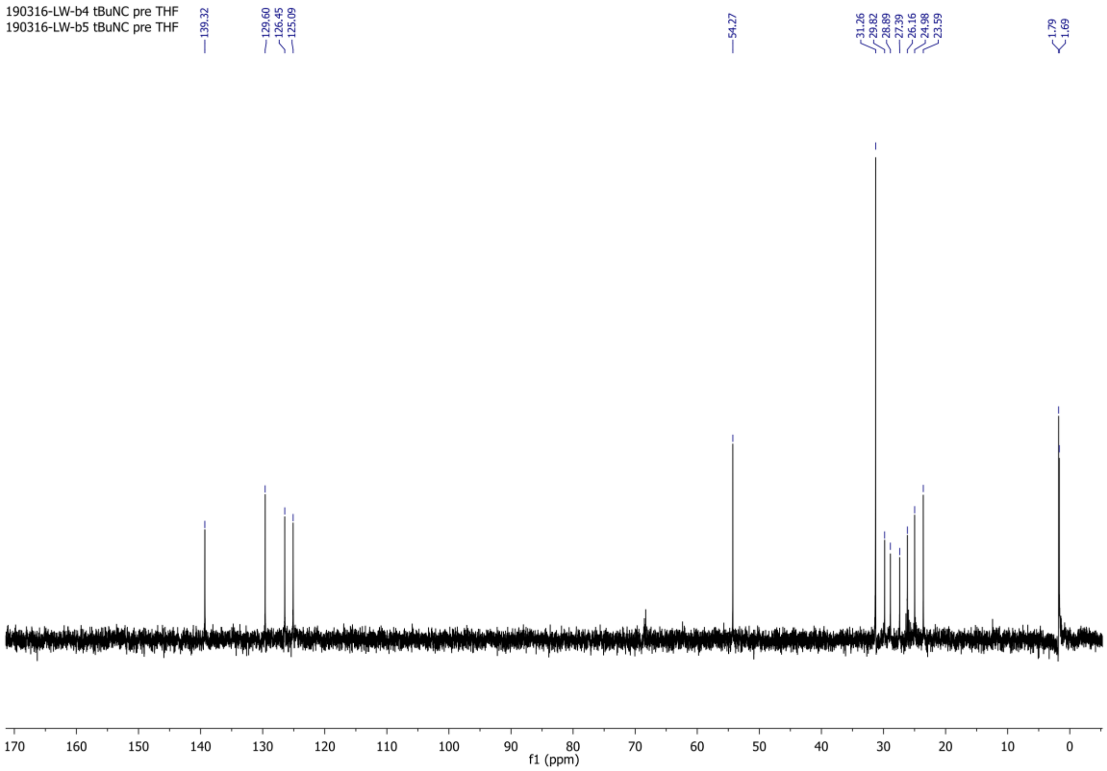

Supplementary Figure 10. $^{13}C\{^{1}H\}$ NMR (DEPT 135) spectrum of **3** in THF-d_8

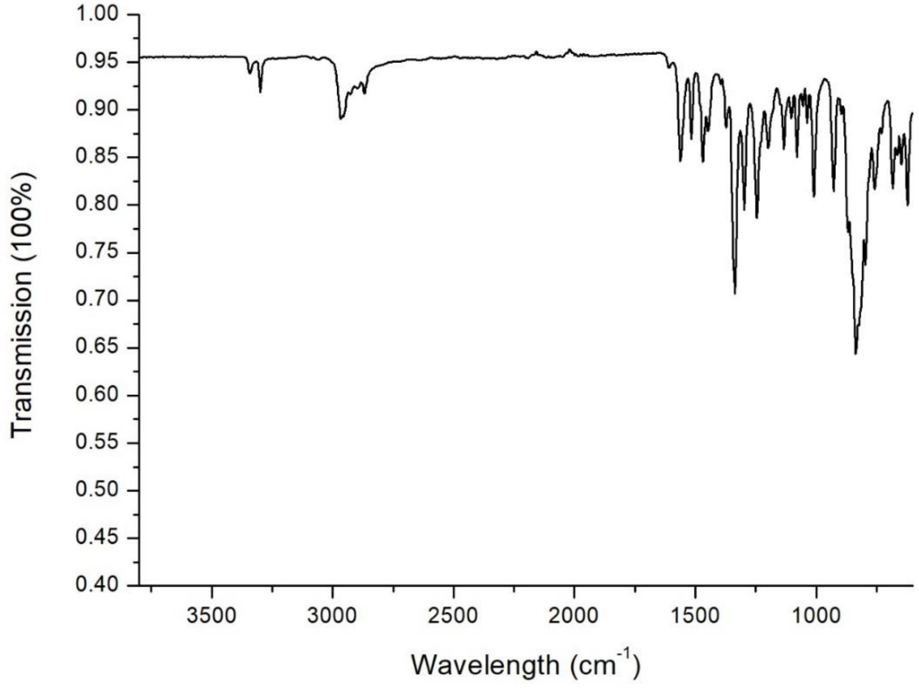

Supplementary Figure 11. FT-IR spectrum of **3**.

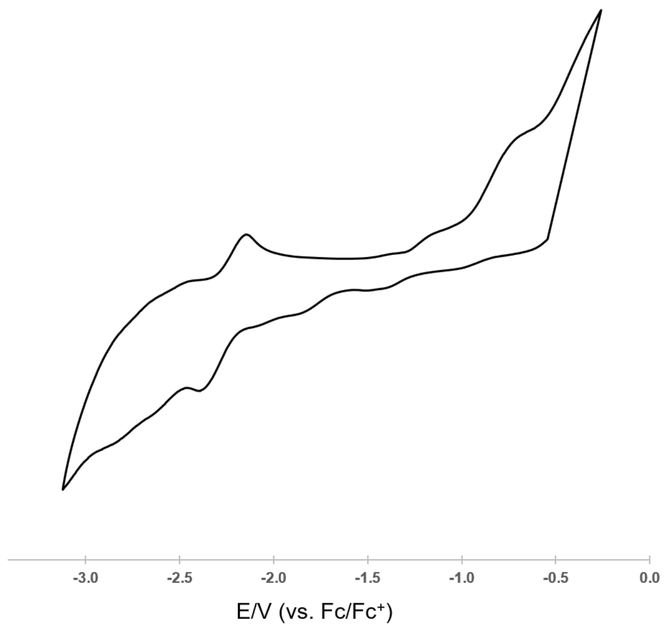

Supplementary Figure 12. Cyclic voltammogram of **3** in THF/0.1 M [^{n}Bu$_4$N][PF$_6$] at room temperature. Scan rate: 100 mVs^{-1}.

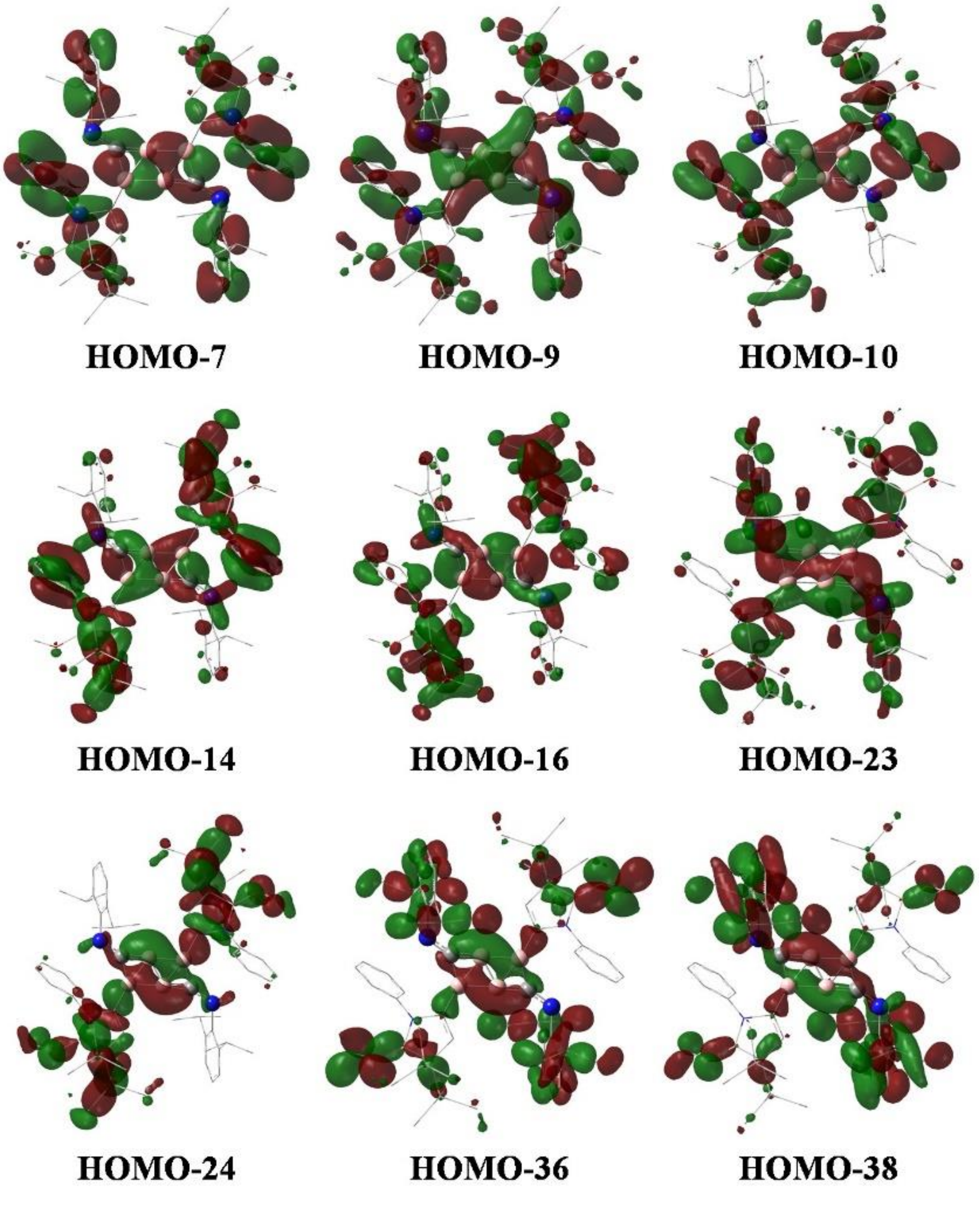

Supplementary Figure 13. Plots of the frontier molecular orbitals of **opt-2’**.

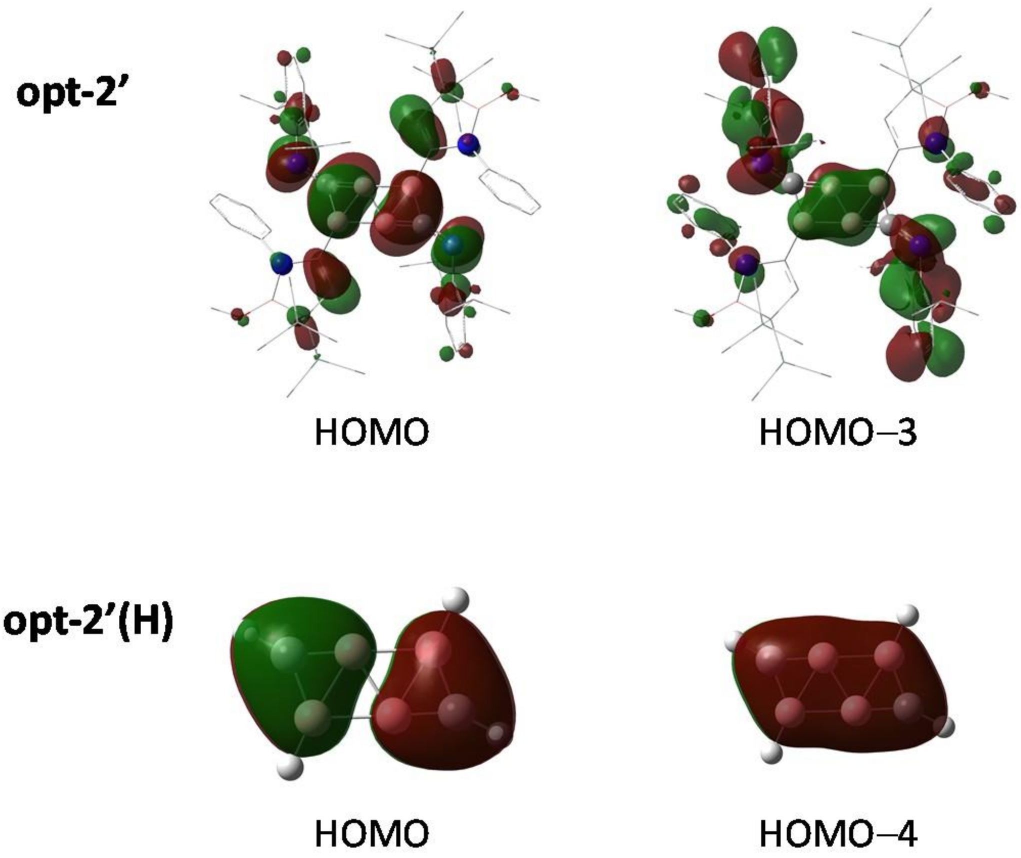

Supplementary Figure 14. Key π MOs of **opt-2’** and **opt-2’(H)**. The HOMO and HOMO–3 of **opt-2’** mainly correspond to the π-type orbitals over the CBB and the central B_4 units, respectively. Likewise, the HOMO of **opt-2’(H)** shows the combination of two CBB π-orbitals, whereas the HOMO–4 of **opt-2’(H)** is dominated by the π-type orbital over the entire C_2B_4 core.

Supplementary Tables:

Supplementary Table 1. X-ray data for compounds **2** and **3**.

Compound	**2**	**$(3)_2$·toluene**
Formula	$C_{70}H_{114}B_6N_4O_2Si_4$	$C_{119}H_{204}B_{12}N_{12}O_4Si_8$
Fw	1220.87	2221.37
Cryst syst	triclinic	triclinic
Space group	P -1	P -1
Size (mm^3)	0.100 x 0.120 x 0.300	0.120 x 0.200 x 0.220
T/K	296(2)	100(2)
a, Å	10.5476(6)	10.4901(2)
b, Å	15.6585(9)	22.5359(3)
c, Å	25.1490(13)	29.3163(5)
α, deg	86.493(3)	89.3060(10)
β, deg	82.659(3)	89.9340(10)
γ, deg	72.064(3)	83.4550(10)
V, A^3	3918.3(4)	6884.82(2)
Z	2	2
d_{calcd} g·cm^{-3}	1.035	1.072
μ, mm^{-1}	1.010	1.115
Refl collected	48744	88572
T_{max}/ T_{min}	0.9060/0.7510	0.7530/0.1962
N_{measd}	13783	24484
[R int]	0.0541	0.0971
R [I>2sigma(I)]	0.0677	0.0701
R_w [I>2sigma(I)]	0.2060	0.1728
GOF	1.104	1.045
Largest diff. peak/ hole[e. Å^{-3}]	0.478/−0.489	0.825/−0.688

Supplementary Table 2: Optimized structure of **opt-2'** (atom, x-, y-, z- positions in Å).

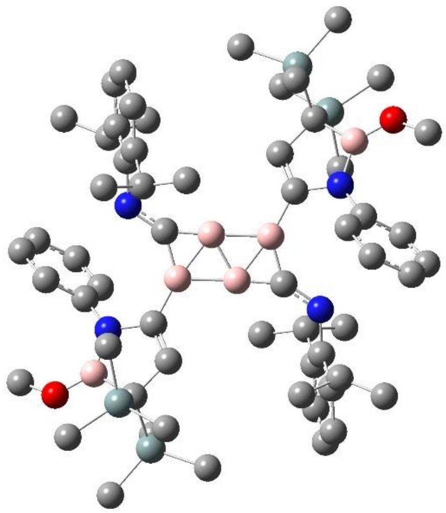

Zero-point correction= 1.373535 (Hartree per Particle)
Thermal correction to Energy= 1.464492
Thermal correction to Enthalpy= 1.465436
Thermal correction to Gibbs Free Energy= 1.236804
Sum of electronic and zero-point Energies= -3940.686764
Sum of electronic and thermal Energies= -3940.595808
Sum of electronic and thermal Enthalpies= -3940.594863
Sum of electronic and thermal Free Energies= -3940.823496

B	-5.042532	1.594227	0.186484
B	-1.268980	0.893014	0.123927
B	0.394120	0.678310	-0.151454
C	-3.183764	3.110052	1.127991
C	-2.594638	3.029573	2.396322
C	-2.187577	4.184317	3.057180
H	-1.744474	4.107531	4.043815
C	-2.360943	5.436291	2.465782
H	-2.052494	6.334246	2.987851
C	-2.924312	5.522398	1.194927
H	-3.051604	6.489010	0.720293
C	-3.322810	4.366112	0.524147
C	-2.820519	0.819298	0.174224
C	-3.579210	-0.206308	-0.290843
H	-3.145762	-1.148164	-0.603022

C	-5.042169	0.130975	-0.410493
C	-6.371743	3.487885	1.169936
H	-5.784836	3.479026	2.091582
H	-7.431164	3.552901	1.423838
H	-6.095576	4.370920	0.587736
C	-5.628309	-0.645049	2.526995
H	-6.115171	-1.362146	3.195915
H	-5.947030	0.356424	2.829907
H	-4.549560	-0.722869	2.686856
C	-5.724878	-2.836838	0.399554
H	-6.296996	-3.454031	1.100529
H	-4.669076	-3.076883	0.544228
H	-6.004652	-3.142511	-0.611344
C	-7.945223	-0.715780	0.527517
H	-8.332645	-1.126871	-0.408289

H	-8.175854	0.351417	0.552365
H	-8.485589	-1.201383	1.346800
C	-5.510254	-1.515448	-3.070961
H	-5.639532	-1.419583	-4.154205
H	-6.321004	-2.147849	-2.699974
H	-4.568680	-2.042439	-2.896836
C	-7.232372	0.970528	-2.543129
H	-7.323178	1.923567	-2.017318
H	-8.043818	0.325602	-2.200150
H	-7.382792	1.153797	-3.612266
C	-4.266765	1.274713	-3.190538
H	-4.514368	1.311757	-4.256463
H	-3.251452	0.882918	-3.094061
H	-4.261588	2.303069	-2.817864
C	-0.245327	1.953918	-0.145825
C	0.974697	3.964308	-0.929380
C	1.289168	3.777154	-2.293171
C	2.390000	4.459449	-2.819651
H	2.648968	4.325226	-3.863994
C	3.144385	5.323684	-2.036993
H	3.991544	5.847599	-2.465860
C	2.807420	5.518592	-0.703906
H	3.401007	6.195713	-0.099884
C	1.727207	4.846921	-0.124455
C	1.407165	5.067747	1.349772
H	0.468915	4.555238	1.564314
C	2.482846	4.438837	2.254505
H	2.583352	3.369567	2.055065
H	2.218687	4.562576	3.309342
H	3.460686	4.902976	2.096065
C	1.207480	6.556184	1.685834
H	2.126876	7.131833	1.545851
H	0.904930	6.671329	2.730942
H	0.433699	7.004910	1.057701
C	0.437804	2.911411	-3.216088
H	-0.352393	2.460745	-2.616662
C	1.247395	1.765063	-3.848062
H	2.040085	2.143626	-4.500439
H	0.595408	1.128166	-4.453464
H	1.713485	1.142919	-3.080384
C	-0.247203	3.766125	-4.300298
H	-0.847147	4.563258	-3.853352
H	-0.909745	3.146311	-4.911666

H	0.483849	4.233634	-4.966564
N	-0.154631	3.289303	-0.360891
H	-0.967736	3.848647	-0.147526
N	-3.669205	1.946361	0.475391
O	-6.195817	2.295762	0.415359
Si	-5.527376	0.200141	-2.271736
Si	-6.088158	-1.011647	0.726161
B	1.268992	-0.893013	-0.123809
B	-0.394095	-0.678370	0.151728
C	0.245359	-1.953972	0.145885
C	2.820530	-0.819342	-0.174143
N	0.154671	-3.289368	0.360820
C	3.579195	0.206335	0.290817
N	3.669239	-1.946410	-0.475203
C	-0.974674	-3.964428	0.929215
H	0.967809	-3.848685	0.147495
H	3.145724	1.148202	0.602934
C	5.042159	-0.130892	0.410496
B	5.042574	-1.594164	-0.186463
C	3.183804	-3.110175	-1.127680
C	-1.289062	-3.777504	2.293047
C	-1.727244	-4.846872	0.124160
Si	5.527455	-0.199914	2.271698
Si	6.087991	1.011760	-0.726306
O	6.195900	-2.295621	-0.415318
C	2.594891	-3.029857	-2.396118
C	3.322616	-4.366146	-0.523590
C	-2.389889	-4.459855	2.819470
C	-0.437617	-2.911907	3.216030
C	-2.807453	-5.518602	0.703545
C	-1.407281	-5.067377	-1.350136
C	5.510144	1.515681	3.070904
C	7.232569	-0.970049	2.543046
C	4.266961	-1.274613	3.190524
C	5.627989	0.645148	-2.527090
C	5.724710	2.836928	-0.399552
C	7.945095	0.715946	-0.527908
C	6.371860	-3.488302	-1.168993
C	2.187830	-4.184677	-3.056845
C	2.924106	-5.522508	-1.194228
H	-2.648812	-4.325800	3.863846
C	-3.144340	-5.323918	2.036689
H	0.352420	-2.461016	2.616561

C	-1.247186	-1.765786	3.848428
C	0.247658	-3.766854	4.299889
H	-3.401096	-6.195600	0.099444
H	-0.468630	-4.555495	-1.564424
C	-2.482454	-4.437259	-2.254643
C	-1.208710	-6.555812	-1.686842
H	5.639396	1.419832	4.154150
H	6.320856	2.148145	2.699935
H	4.568532	2.042595	2.896741
H	7.323458	-1.923129	2.017330
H	8.043897	-0.325052	2.199922
H	7.383098	-1.153169	3.612193
H	4.514552	-1.311687	4.256449
H	3.251627	-0.882861	3.094052
H	4.261826	-2.302950	2.817798
H	6.114838	1.362239	-3.196029
H	5.946705	-0.356331	-2.829983
H	4.549237	0.722971	-2.686918
H	6.296698	3.454200	-1.100563
H	4.668881	3.076981	-0.544010
H	6.004662	3.142507	0.611326
H	8.332658	1.127093	0.407817
H	8.175749	-0.351247	-0.552744
H	8.485328	1.201510	-1.347305

H	5.785012	-3.480141	-2.090679
H	7.431297	-3.553515	-1.422772
H	6.095656	-4.370899	-0.586136
H	1.744906	-4.108019	-4.043571
C	2.360964	-5.436563	-2.465198
H	3.051212	-6.489052	-0.719406
H	-3.991503	-5.847872	2.465498
H	-2.039711	-2.144549	4.500886
H	-0.595133	-1.128959	4.453834
H	-1.713491	-1.143524	3.080972
H	0.847630	-4.563786	3.852620
H	0.910215	-3.147151	4.911353
H	-0.483256	-4.234647	4.966108
H	-2.581981	-3.367948	-2.054926
H	-2.218490	-4.560959	-3.309535
H	-3.460692	-4.900569	-2.096226
H	-2.128600	-7.130804	-1.547407
H	-0.905981	-6.670686	-2.731927
H	-0.435457	-7.005468	-1.058724
H	2.052503	-6.334580	-2.987157
H	-2.480598	2.058947	2.863713
H	-3.754384	4.425729	-0.468403
H	2.481028	-2.059298	-2.863692
H	3.754007	-4.425630	0.469047

Supplementary Table 3: Optimized structure of **opt-2’(H)** (atom, x-, y-, z- positions in Å).

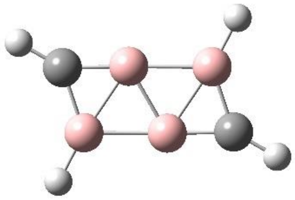

Zero-point correction= 0.063442 (Hartree per Particle)
Thermal correction to Energy= 0.069028
Thermal correction to Enthalpy= 0.069972
Thermal correction to Gibbs Free Energy= 0.035428
Sum of electronic and zero-point Energies= -177.943919
Sum of electronic and thermal Energies= -177.938333
Sum of electronic and thermal Enthalpies= -177.937389
Sum of electronic and thermal Free Energies= -177.971933

B	1.237270	-0.901212	0.000094
B	0.515885	0.652458	-0.000138
C	1.906437	0.418807	0.000069
B	-1.237170	0.901233	-0.000099
B	-0.515944	-0.652599	-0.000026
C	-1.906469	-0.418767	0.000102
H	-1.610177	2.024618	0.000098
H	1.610309	-2.024582	-0.000495
H	-2.904312	-0.812970	0.000037
H	2.904166	0.813290	0.000184

Supplementary Table 4: Optimized structure of **opt-3'** (*trans*) (atom, x-, y-, z- positions in Å).

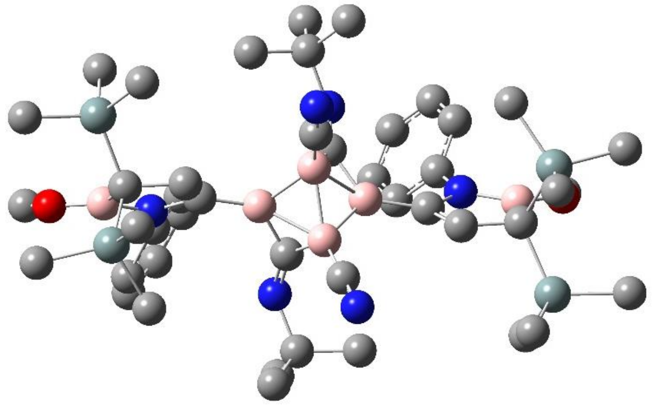

Zero-point correction= 1.119745 (Hartree per Particle)
Thermal correction to Energy= 1.199112
Thermal correction to Enthalpy= 1.200056
Thermal correction to Gibbs Free Energy= 1.000099
Sum of electronic and zero-point Energies= -3507.260322
Sum of electronic and thermal Energies= -3507.180955
Sum of electronic and thermal Enthalpies= -3507.180011
Sum of electronic and thermal Free Energies= -3507.379967

C	-0.475468	0.521511	-1.293615
C	0.475494	0.520587	1.293724
C	-2.854705	-0.128751	0.005120
C	-3.465348	-1.286270	0.366310
H	-2.909348	-2.160416	0.678395
C	2.854737	-0.128709	-0.005407
C	3.465418	-1.285921	-0.367520
H	2.909449	-2.159777	-0.680483
C	0.426769	-2.057133	-1.742711
C	-0.426829	-2.058363	1.741025
C	0.047660	1.248703	-3.668873
C	-0.130069	2.621769	-4.333043
H	-1.186930	2.897837	-4.393011
H	0.268608	2.599907	-5.348906
H	0.398525	3.399711	-3.775192
C	-0.709087	0.171490	-4.466417
H	-0.588946	-0.813029	-4.012261
H	-0.323762	0.120732	-5.488482

H	-1.776036	0.403663	-4.513988
C	1.538276	0.905071	-3.576847
H	2.084892	1.673017	-3.024528
H	1.958352	0.849825	-4.584059
H	1.707545	-0.056739	-3.095081
C	-0.047606	1.246172	3.669489
C	-1.538204	0.902542	3.577247
H	-2.084844	1.670782	3.025366
H	-1.958284	0.846712	4.584426
H	-1.707440	-0.058998	3.094934
C	0.130048	2.618815	4.334552
H	1.186894	2.894892	4.394753
H	-0.268675	2.596277	5.350382
H	-0.398550	3.397093	3.777179
C	0.709172	0.168474	4.466348
H	0.589046	-0.815757	4.011560
H	0.323867	0.117058	5.488388
H	1.776119	0.400639	4.514046

C	-4.959330	-1.253953	0.221953
C	-6.722095	2.196867	-0.434614
H	-6.049871	2.794444	0.186326
H	-7.741615	2.304617	-0.060064
H	-6.683693	2.577732	-1.458574
C	-3.508800	2.136120	-0.838735
C	-2.691432	2.974480	-0.066675
C	-2.373488	4.256364	-0.507973
H	-1.744693	4.890662	0.106971
C	-2.871807	4.732200	-1.721019
H	-2.631692	5.734091	-2.057014
C	-3.679433	3.903240	-2.497837
H	-4.065419	4.255757	-3.447959
C	-3.983170	2.612479	-2.069619
C	-5.470368	-0.000506	3.009066
H	-5.993880	0.877478	2.620049
H	-4.404878	0.235783	3.066715
H	-5.827678	-0.172330	4.029629
C	-7.653076	-1.771591	1.818292
H	-8.090450	-1.734166	2.821534
H	-7.922637	-2.736030	1.380642
H	-8.116219	-0.984598	1.218268
C	-5.024328	-3.019506	2.827225
H	-5.463510	-3.112166	3.826289
H	-3.942887	-2.917870	2.951578
H	-5.208950	-3.958141	2.299517
C	-7.275284	-2.217384	-1.702262
H	-7.485771	-1.162026	-1.891044
H	-7.982137	-2.564555	-0.946357
H	-7.472552	-2.775956	-2.623196
C	-5.219795	-4.272930	-0.670758
H	-5.919953	-4.585088	0.108509
H	-4.205810	-4.453387	-0.303544
H	-5.377195	-4.927661	-1.534156
C	-4.389695	-2.139680	-2.698134
H	-4.690756	-2.794616	-3.522169
H	-3.333627	-2.329324	-2.492529
H	-4.482226	-1.106916	-3.047324
C	4.959371	-1.253789	-0.222815
C	3.508737	2.135575	0.840030
C	3.982646	2.611269	2.071351
C	3.678901	3.901855	2.500080
H	4.064556	4.253853	3.450529

C	2.871688	4.731301	1.723346
H	2.631571	5.733062	2.059727
C	2.373774	4.256108	0.509885
H	1.745298	4.890778	-0.105003
C	2.691736	2.974401	0.068088
C	5.219351	-4.273447	0.667593
H	5.920173	-4.585147	-0.111259
H	4.205677	-4.453406	0.299279
H	5.375786	-4.928883	1.530630
C	7.274398	-2.218786	1.701805
H	7.471817	-2.780007	2.621096
H	7.484143	-1.163862	1.893837
H	7.981644	-2.563170	0.945005
C	4.388563	-2.141896	2.696490
H	3.332567	-2.331556	2.490564
H	4.480894	-1.109393	3.046504
H	4.689581	-2.797421	3.520081
C	5.025338	-3.017522	-2.829318
H	5.464584	-3.109131	-3.828451
H	3.943882	-2.915973	-2.953641
H	5.210116	-3.956620	-2.302500
C	7.653676	-1.770048	-1.818595
H	7.923237	-2.735067	-1.382218
H	8.116398	-0.983814	-1.217258
H	8.091460	-1.731099	-2.821601
C	5.471057	0.001578	-3.008838
H	5.994466	0.879345	-2.619193
H	4.405551	0.237838	-3.066424
H	5.828468	-0.169568	-4.029477
N	0.626852	-2.976372	-2.416450
N	-0.626914	-2.978042	2.414171
N	-0.553498	1.364806	-2.305391
N	0.553578	1.363166	2.306098
N	-3.851472	0.834998	-0.399962
N	3.851462	0.834669	0.400693
O	-6.400585	0.813175	-0.387964
Si	-5.781846	-1.533395	1.939708
Si	-5.475132	-2.471208	-1.183415
Si	5.474512	-2.472159	1.181868
Si	5.782460	-1.532003	-1.940444
C	6.722436	2.196216	0.437581
H	6.050537	2.794765	-0.182765
H	7.742090	2.304208	0.063469

H	6.683837	2.575736	1.462034		B	5.166408	0.238911	0.246200
O	6.400628	0.812666	0.389039		H	1.220568	2.119323	2.206292
B	-1.319283	0.029292	-0.141347		H	-1.220397	2.120973	-2.205029
B	0.171837	-0.806384	-0.880130		H	-4.584027	1.957660	-2.689559
B	-0.171851	-0.806990	0.879338		H	-2.315071	2.617304	0.883699
B	1.319306	0.029169	0.141081		H	4.583159	1.956070	2.691217
B	-5.166407	0.239143	-0.245765		H	2.315707	2.617727	-0.882609

Supplementary Table 5: Optimized structure of **opt-2’’** (atom, x-, y-, z- positions in Å).

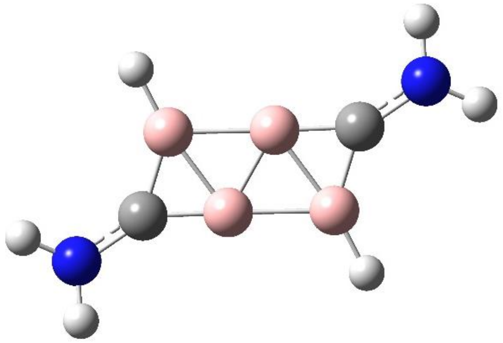

Zero-point correction= 0.098620 (Hartree per Particle)
Thermal correction to Energy= 0.107151
Thermal correction to Enthalpy= 0.108095
Thermal correction to Gibbs Free Energy= 0.066746
Sum of electronic and zero-point Energies= -288.661304
Sum of electronic and thermal Energies= -288.652773
Sum of electronic and thermal Enthalpies= -288.651829
Sum of electronic and thermal Free Energies= -288.693178

B	1.006473	1.160686	-0.001647		C	-1.972574	-0.037479	-0.005111
B	0.639705	-0.479563	0.004323		N	-3.304250	0.222946	-0.057097
C	1.972596	0.037661	0.004990		H	-3.944350	-0.520561	0.172401
N	3.304249	-0.223306	0.057159		H	-3.621708	1.146260	0.191362
H	3.944411	0.520105	-0.172551		H	3.621206	-1.146440	-0.192660
B	-1.006283	-1.160494	0.001729		H	-1.071160	-2.343136	0.009465
B	-0.639751	0.479740	-0.004004		H	1.070759	2.343362	-0.009736

Supplementary Table 6: Optimized structure of **opt-2'T** (atom, x-, y-, z- positions in Å).

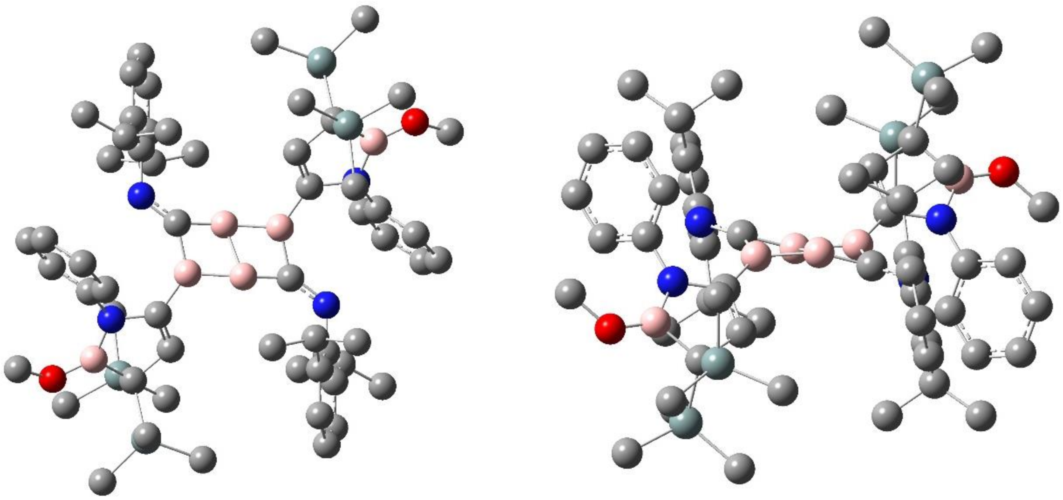

View from the top (*left*) and sideways view

Zero-point correction= 1.371959 (Hartree per Particle)
Thermal correction to Energy= 1.463098
Thermal correction to Enthalpy= 1.464042
Thermal correction to Gibbs Free Energy= 1.230839
Sum of electronic and zero-point Energies= -3940.639837
Sum of electronic and thermal Energies= -3940.548698
Sum of electronic and thermal Enthalpies= -3940.547754
Sum of electronic and thermal Free Energies= -3940.780957

B	-5.123533	1.416176	0.235395
B	-1.318133	0.753048	0.312611
B	0.378698	0.621623	-0.482762
C	-3.309647	2.946158	1.243639
C	-2.728771	2.844120	2.515147
C	-2.346645	3.988410	3.208329
H	-1.909663	3.893466	4.196130
C	-2.537007	5.252541	2.648006
H	-2.247988	6.141953	3.195414
C	-3.093487	5.361376	1.375850
H	-3.234882	6.337431	0.925120
C	-3.466573	4.215700	0.672119
C	-2.881604	0.690659	0.244007
C	-3.611473	-0.336189	-0.265454
H	-3.150704	-1.259330	-0.592912
C	-5.082217	-0.033908	-0.392026
C	-6.508358	3.255077	1.243937
H	-5.938030	3.230930	2.175738

H	-7.573191	3.292355	1.480366
H	-6.239797	4.160064	0.692565
C	-5.621552	-0.862886	2.542566
H	-6.085316	-1.600324	3.205853
H	-5.962797	0.126246	2.861011
H	-4.539850	-0.915777	2.692663
C	-5.659510	-3.032234	0.396979
H	-6.230364	-3.674460	1.076283
H	-4.600170	-3.242145	0.563765
H	-5.906804	-3.331171	-0.624350
C	-7.950672	-0.991022	0.557307
H	-8.329362	-1.403915	-0.381343
H	-8.221051	0.066440	0.596873
H	-8.467546	-1.506227	1.373751
C	-5.495547	-1.658819	-3.073892
H	-5.638265	-1.551820	-4.154423
H	-6.276526	-2.329592	-2.706361
H	-4.531208	-2.148065	-2.915356

C	-7.310048	0.750134	-2.503811
H	-7.436074	1.689733	-1.961294
H	-8.094002	0.067840	-2.168826
H	-7.472154	0.945119	-3.569167
C	-4.356882	1.176973	-3.150957
H	-4.599184	1.215070	-4.218055
H	-3.327299	0.825163	-3.050527
H	-4.396200	2.200710	-2.767468
C	-0.343835	1.850393	-0.142310
C	0.711742	3.945048	-0.932524
C	0.956213	3.837092	-2.316663
C	2.000827	4.593143	-2.857969
H	2.209936	4.525012	-3.919566
C	2.762075	5.443361	-2.067478
H	3.567604	6.020019	-2.508554
C	2.483423	5.562245	-0.711485
H	3.078294	6.233959	-0.103811
C	1.457494	4.823007	-0.117668
C	1.192048	4.954841	1.378995
H	0.220249	4.506250	1.589857
C	2.234108	4.169623	2.197186
H	2.227000	3.111195	1.927757
H	2.019886	4.246677	3.267557
H	3.242742	4.556120	2.025078
C	1.120154	6.420085	1.841915
H	2.085002	6.927243	1.753197
H	0.824846	6.466295	2.894068
H	0.389199	6.986455	1.259204
C	0.084417	2.996394	-3.243649
H	-0.616817	2.432525	-2.629378
C	0.902810	1.975523	-4.052739
H	1.605088	2.466195	-4.732905
H	0.236387	1.353742	-4.657631
H	1.470010	1.318742	-3.389585
C	-0.748960	3.900941	-4.173364
H	-1.359296	4.603069	-3.599028
H	-1.419990	3.296844	-4.790974
H	-0.110188	4.485334	-4.842277
N	-0.346369	3.177625	-0.334588
H	-1.140003	3.697560	0.019513
N	-3.762124	1.789922	0.555924
O	-6.295191	2.088956	0.459454
Si	-5.575322	0.042605	-2.247204

Si	-6.082234	-1.221585	0.740201
B	1.318197	-0.752796	-0.312670
B	-0.378637	-0.621449	0.482683
C	0.343821	-1.850203	0.141983
C	2.881665	-0.690413	-0.243643
N	0.346387	-3.177487	0.333853
C	3.611418	0.336219	0.266400
N	3.762240	-1.789570	-0.555781
C	-0.711710	-3.945236	0.931393
H	1.140022	-3.697269	-0.020479
H	3.150579	1.259229	0.594125
C	5.082124	0.033851	0.393257
B	5.123568	-1.415992	-0.234721
C	3.309865	-2.945517	-1.244052
C	-0.956344	-3.837836	2.315545
C	-1.457264	-4.822984	0.116123
Si	5.574650	-0.043417	2.248554
Si	6.082508	1.221866	-0.738293
O	6.295268	-2.088742	-0.458675
C	2.729229	-2.842926	-2.515626
C	3.466609	-4.215300	-0.673017
C	-2.000887	-4.594262	2.856457
C	-0.084752	-2.997377	3.242932
C	-2.483126	-5.562622	0.709559
C	-1.191738	-4.954079	-1.380591
C	5.493835	1.657533	3.076121
C	7.309574	-0.750367	2.505423
C	4.356354	-1.178744	3.151297
C	5.622060	0.864019	-2.540892
C	5.660024	3.032433	-0.394364
C	7.950892	0.990955	-0.555228
C	6.508613	-3.254499	-1.243652
C	2.347149	-3.986909	-3.209341
C	3.093576	-5.360662	-1.377283
H	-2.210109	-4.526566	3.918061
C	-2.761918	-5.444311	2.065571
H	0.616245	-2.432919	2.628933
C	-0.903400	-1.977246	4.052685
C	0.749007	-3.902194	4.172041
H	-3.077840	-6.234186	0.101566
H	-0.219874	-4.505493	-1.591174
C	-2.233675	-4.168293	-2.198395
C	-1.119996	-6.419074	-1.844314

H	5.636362	1.550031	4.156628
H	6.274539	2.328919	2.709136
H	4.529275	2.146365	2.917636
H	7.436202	-1.689604	1.962418
H	8.093373	-0.067547	2.171148
H	7.471340	-0.945902	3.570730
H	4.598076	-1.217044	4.218518
H	3.326640	-0.827451	3.050397
H	4.396444	-2.202342	2.767515
H	6.085962	1.601744	-3.203762
H	5.963313	-0.124977	-2.859747
H	4.540384	0.917032	-2.691131
H	6.231142	3.674867	-1.073250
H	4.600756	3.242584	-0.561299
H	5.907138	3.330856	0.627158
H	8.329569	1.403751	0.383472
H	8.221094	-0.066551	-0.594813
H	8.467933	1.506104	-1.371602
H	5.938563	-3.229874	-2.175608
H	7.573515	-3.291717	-1.479784
H	6.239847	-4.159740	-0.692793
H	1.910351	-3.891537	-4.197183
C	2.537325	-5.251277	-2.649494
H	3.234825	-6.336911	-0.926927
H	-3.567383	-6.021278	2.506356
H	-1.605306	-2.468530	4.732799
H	-0.237107	-1.355460	4.657714
H	-1.471050	-1.320402	3.389981
H	1.359595	-4.603713	3.597229
H	1.419831	-3.298235	4.790010
H	0.110484	-4.487272	4.840597
H	-2.226440	-3.110004	-1.928421
H	-2.019454	-4.244823	-3.268804
H	-3.242357	-4.554763	-2.026492
H	-2.084920	-6.926150	-1.755975
H	-0.824596	-6.464729	-2.896463
H	-0.389171	-6.985866	-1.261853
H	2.248343	-6.140452	-3.197308
H	-2.599215	1.863635	2.957043
H	-3.891626	4.293699	-0.321977
H	2.599822	-1.862251	-2.957147
H	3.891477	-4.293727	0.321125

Supplementary Table 7: Optimized structure of **III** (atom, x-, y-, z- positions in Å).

Zero-point correction= 0.037398 (Hartree per Particle)
Thermal correction to Energy= 0.046174
Thermal correction to Enthalpy= 0.047118
Thermal correction to Gibbs Free Energy= 0.003289
Sum of electronic and zero-point Energies= -414.283123
Sum of electronic and thermal Energies= -414.274347
Sum of electronic and thermal Enthalpies= -414.273403
Sum of electronic and thermal Free Energies= -414.317232

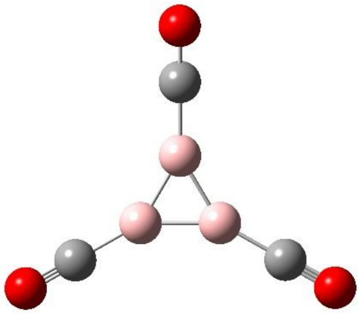

B	0.000000	0.900608	0.000000
B	0.779950	-0.450304	0.000000
B	-0.779950	-0.450304	0.000000
C	2.062446	-1.190754	0.000000

C	0.000000	2.381507	0.000000
C	-2.062446	-1.190754	0.000000
O	3.037878	-1.753920	0.000000
O	0.000000	3.507840	0.000000
O	-3.037878	-1.753920	0.000000

Supplementary Table 8: Optimized structure of **IV** (atom, x-, y-, z- positions in Å).

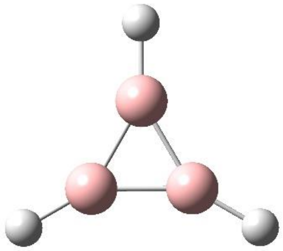

Zero-point correction= 0.031380 (Hartree per Particle)
Thermal correction to Energy= 0.035002
Thermal correction to Enthalpy= 0.035947
Thermal correction to Gibbs Free Energy= 0.007332
Sum of electronic and zero-point Energies= -76.109501
Sum of electronic and thermal Energies= -76.105879
Sum of electronic and thermal Enthalpies= -76.104935
Sum of electronic and thermal Free Energies= -76.133549

B	0.000000	-0.928058	0.000000
B	-0.803485	0.464976	0.000000
B	0.804528	0.463249	0.000000
H	-0.006000	-2.170842	0.000000
H	-1.880344	1.084027	0.000000
H	1.881133	1.085979	0.000000

Supplementary Table 9: Optimized structure of **V** (atom, x-, y-, z- positions in Å).

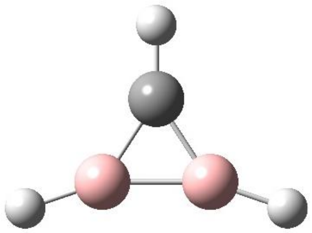

Zero-point correction= 0.036815 (Hartree per Particle)
Thermal correction to Energy= 0.040250
Thermal correction to Enthalpy= 0.041194
Thermal correction to Gibbs Free Energy= 0.012956
Sum of electronic and zero-point Energies= -89.565953
Sum of electronic and thermal Energies= -89.562519
Sum of electronic and thermal Enthalpies= -89.561574
Sum of electronic and thermal Free Energies= -89.589812

B	0.798045	-0.468637	0.000000
B	-0.798001	-0.468762	0.000000
H	1.939108	-0.859257	0.000000
H	-1.939308	-0.859304	0.000000
H	-0.000018	1.851464	0.000000
C	0.000000	0.759016	0.000000

Supplementary Table 10. Experimental and calculated ^{11}B NMR chemical shifts of **2** and **3**.

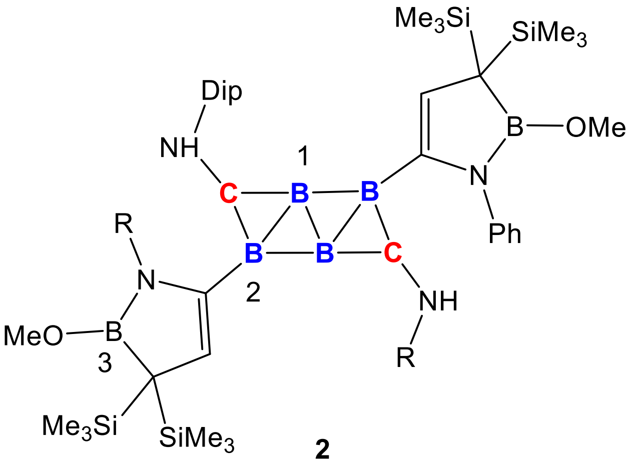

2

δ (^{11}B)[ppm]	B1	B2	B3
exp. (R=Dip)	16.5	11.3	35.6
cal. (R=Ph)	14.4	9.8	36.2

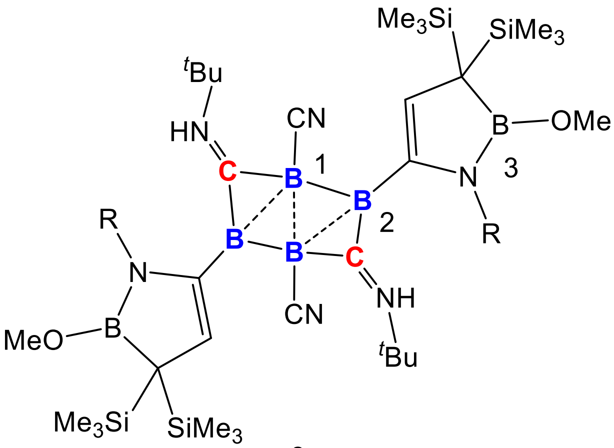

3

δ (^{11}B)[ppm]	B1	B2	B3
exp. (R=Dip)	−12.2	12.2	36.8
cal. (R=Ph)	−12.3	12.3	36.9

Supplementary Table 11. Selected data of NPA charges of **opt-2'** at B3LYP/6-311G** level of theory.

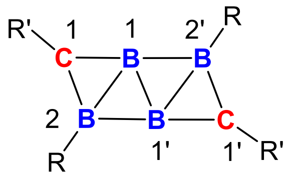

opt-2'

Summary of Natural Population Analysis:

Atom	No	Natural Charge	Natural Population: Core	Valence	Rydberg	Total
C	1	-0.28131	1.99804	4.26019	0.02307	6.28131
B	1	0.22532	1.99753	2.75601	0.02113	4.77468
B	2	0.29439	1.99824	2.69073	0.01665	4.70561
B	1'	0.22527	1.99753	2.75606	0.02113	4.77473
C	1'	-0.28132	1.99804	4.26020	0.02307	6.28132
B	2'	0.29443	1.99824	2.69069	0.01665	4.70557

Supplementary Table 12. Selected data of NPA charges of **opt-3'** at B3LYP/6-311G** level of theory.

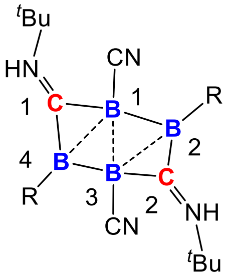

opt-3'

Summary of Natural Population Analysis:

Atom	No	Natural Charge	Natural Population: Core	Valence	Rydberg	Total
C	1	-0.10206	1.99841	4.07115	0.03250	6.10206
B	1	-0.04818	1.99787	3.02979	0.02052	5.04818
B	2	0.48606	1.99823	2.48971	0.02600	4.51394
C	2	-0.10208	1.99841	4.07117	0.03250	6.10208
B	3	-0.04812	1.99787	3.02973	0.02052	5.04812
B	4	0.48605	1.99823	2.48972	0.02600	4.51395

Supplementary Table 13. Wiberg bond index (WBI) values of **opt-2'** at B3LYP/6-311G** level of theory.

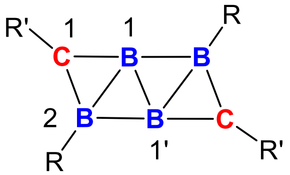

opt-2'

	B1-C1	B2-C1	B1-B2	B1-B1'	B2-B1'
WBI	1.3091	1.1069	0.6271	0.8041	0.5933

Supplementary Table 14. Selected metric parameters [distances (Å) and angles (°)] of **opt-2’** and **opt-2’(H).**

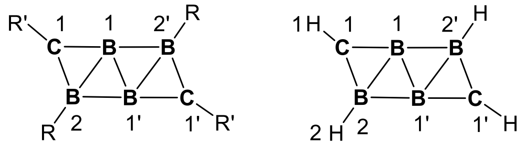

	**opt-2'**		**opt-2'(H)**	
Distance (Å)	B(1)—C(1)	1.427	B(1)—C(1)	1.410
	B(2)—C(1)	1.499	B(2)—C(1)	1.480
	B(1)—B(2)	1.699	B(1)—B(2)	1.713
	B(1)—B(1')	1.598	B(1)—B(1')	1.664
	B(1')—B(2)	1.799	B(1')—B(2)	1.771
Bond angle (°)	B(2)-B(1')-C(1')	177.3	B(2)-B(1')-C(1')	178.5
	B(2)-C(1)-B(1)	71.0	B(2)-C(1)-B(1)	72.7
	B(2)-B(1)-C(1)	56.5	B(2)-B(1)-C(1)	55.6
	B(1)-B(2)-C(1)	52.5	B(1)-B(2)-C(1)	51.8
	B(2)-B(1)-B(1')	66.0	B(2)-B(1)-B(1')	63.2
	B(2)-B(1')-B(1)	59.7	B(2)-B(1')-B(1)	59.8
	B(1')-B(2)-B(1)	54.3	B(1')-B(2)-B(1)	57.0
Dihedral angle (°)	R'-C(1)-B(2)-R	6.4	H(1)-C(1)-B(2)-H(2)	0.0
	C(1)-B(2)-B(1)-B(1')	178.1	C(1)-B(2)-B(1)-B(1')	180.0

While **opt-2’(H)** has perfectly planar geometry, the C_2B_4 unit and the corresponding four peripheral atoms of **opt-2’** deviate slightly from planarity with the R’-C(1)-B(2)-R and C(1)-B(2)-B(1)-B(1’) dihedral angles of 6.4° and 178.1°, respectively. The B-C distances [B(1)-C(1): 1.427 Å and B(2)-C(1): 1.499 Å for the CBB rings] of **opt-2’** are slightly longer than those [B(1)-C(1): 1.414 Å and B(2)-C(1): 1.480 Å for the CBB rings] observed in **opt-2’(H)**. The B-B distances of B(1)-B(2) (1.699 Å) and B(1)-B(1’) (1.598 Å) in **opt-2’** are shorter than those (1.713 Å and 1.664 Å) found in **opt-2’(H)**, whereas the B(1’)-B(2) distance (1.799 Å) is slightly longer as compared to that (1.771 Å) observed in **opt-2’(H)**. These structural differences between **opt-2’** and **opt-2’(H)** indicates the influence of the bulky substituents in **opt-2’** on the geometric features of $C_2B_4R_4$ molecule. (*4*)

Supplementary Table 15. Natural bond orbital analysis of **opt-2'** at B3LYP/6-311G** level of theory.

NATURAL BOND ORBITAL ANALYSIS:

Cycle	Occ. Thresh.	Occupancies Lewis	Non-Lewis	Lewis Structure CR	BD	3C	LP	Low occ (L)	High occ (NL)	Dev
1(1)	1.90	554.60404	13.39596	90	170	20	4	12	17	1.11
2(2)	1.90	555.74862	12.25138	90	170	21	3	14	16	0.77
3(3)	1.90	555.24834	12.75166	90	170	20	4	14	18	0.77
4(4)	1.90	555.73408	12.26592	90	170	20	4	14	17	0.77
5(5)	1.90	555.24834	12.75166	90	170	20	4	14	18	0.77
6(6)	1.90	555.73408	12.26592	90	170	20	4	14	17	0.77
7(7)	1.90	555.24834	12.75166	90	170	20	4	14	18	0.77
8(8)	1.90	555.73408	12.26592	90	170	20	4	14	17	0.77
9(9)	1.90	555.24834	12.75166	90	170	20	4	14	18	0.77
10(1)	1.80	556.06539	11.93461	90	174	16	4	6	16	0.45
11(2)	1.80	556.09960	11.90040	90	174	16	4	6	15	0.44
12(3)	1.80	556.10732	11.89268	90	174	16	4	6	15	0.44
13(4)	1.80	556.10732	11.89268	90	174	16	4	6	15	0.44
14(1)	1.70	557.47292	10.52708	90	172	16	6	4	15	0.44
15(2)	1.70	557.10815	10.89185	90	172	16	6	4	17	0.43
16(3)	1.70	556.72048	11.27952	90	172	16	6	4	19	0.43
17(4)	1.70	557.10562	10.89438	90	172	16	6	4	18	0.43
18(5)	1.70	557.50439	10.49561	90	172	16	6	4	16	0.42
19(6)	1.70	557.45789	10.54211	90	172	16	6	4	17	0.45
20(7)	1.70	557.47039	10.52961	90	172	16	6	4	16	0.44
21(8)	1.70	557.07229	10.92771	90	172	16	6	4	18	0.45
22(9)	1.70	557.07352	10.92648	90	172	16	6	4	18	0.44
23(1)	1.60	556.07058	11.92942	90	179	4	11	1	24	0.90
24(2)	1.60	556.07058	11.92942	90	179	4	11	1	24	0.90
25(1)	1.50	550.07677	17.92323	90	177	4	13	6	33	0.91
26(2)	1.50	551.07694	16.92306	90	176	5	13	5	32	0.91
27(3)	1.50	551.75478	16.24522	90	175	5	14	5	31	0.90
28(4)	1.50	551.75478	16.24522	90	175	5	14	5	31	0.90
29(1)	1.70	557.50439	10.49561	90	172	16	6	4	16	0.42

Supplementary Table 16. 3-center-2-electron bonding analysis of **opt-2’**.

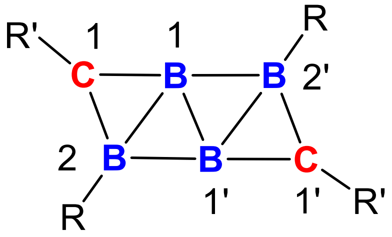

opt-2'

```
(Occupancy)   Bond orbital/ Coefficients/ Hybrids
 ---------------------------------------------------------------------------------

5. (1.88343) 3C ( 1) B 2’ - B 1’ - C 1’
                  ( 25.47%)   0.5047* B 2’  s(  0.13%)p99.99( 99.68%)d 1.41(  0.18%)
                                             -0.0002  0.0349  0.0094  0.0020  0.0747
                                              0.0009  0.0022 -0.0618  0.0117 -0.0039
                                              0.9935  0.0106  0.0117  0.0070 -0.0372
                                              0.0147 -0.0021  0.0138
                  ( 28.27%)   0.5317* B 1’  s(  0.05%)p99.99( 99.33%)d12.30(  0.62%)
                                             -0.0002  0.0215  0.0058  0.0019  0.2507
                                              0.0018 -0.0000 -0.0799 -0.0117  0.0009
                                              0.9610  0.0204  0.0023 -0.0028  0.0690
                                              0.0163  0.0219 -0.0255
                  ( 46.26%)   0.6801* C 1’  s(  0.08%)p99.99( 99.87%)d 0.62(  0.05%)
                                              0.0002  0.0278 -0.0056  0.0008  0.2214
                                              0.0094 -0.0013 -0.1539 -0.0029  0.0008
                                              0.9622  0.0101 -0.0052 -0.0064  0.0064
                                             -0.0190 -0.0035 -0.0065
6. (1.90888) 3C ( 1) B 2’ - B 1’ - B 1
                  ( 35.24%)   0.5936* B 2’  s( 37.13%)p 1.69( 62.84%)d 0.00(  0.03%)
                                              0.0007 -0.6089 -0.0221  0.0015  0.5011
                                             -0.0186 -0.0023  0.6130 -0.0269 -0.0025
                                              0.0222  0.0055  0.0014 -0.0112 -0.0003
                                             -0.0006  0.0019  0.0112
                  ( 25.85%)   0.5084* B 1’  s( 14.98%)p 5.64( 84.56%)d 0.03(  0.46%)
                                              0.0010 -0.3861 -0.0273  0.0033 -0.8500
                                              0.0031  0.0031  0.2435 -0.0318 -0.0004
                                              0.2506 -0.0012 -0.0014  0.0206  0.0292
                                             -0.0097 -0.0315  0.0468
                  ( 38.91%)   0.6238* B 1   s( 43.30%)p 1.31( 56.56%)d 0.00(  0.14%)
                                             -0.0002 -0.6580  0.0058 -0.0010 -0.4542
                                              0.0219  0.0038 -0.5964  0.0142 -0.0031
                                              0.0541  0.0022 -0.0018 -0.0013 -0.0014
                                             -0.0077  0.0292  0.0218

10. (1.90888) 3C ( 1) B 1’ - B 2 - B 1
```

```
                     ( 38.91%)   0.6238* B 1'  s( 43.30%)p 1.31( 56.56%)d 0.00(  0.14%)
                                                -0.0002 -0.6580  0.0058 -0.0010  0.4542
                                                -0.0219 -0.0038  0.5964 -0.0142  0.0031
                                                -0.0541 -0.0022  0.0018 -0.0013 -0.0014
                                                -0.0077  0.0292  0.0218
                     ( 35.24%)   0.5936* B 2   s( 37.13%)p 1.69( 62.84%)d 0.00(  0.03%)
                                                 0.0007 -0.6090 -0.0221  0.0015 -0.5011
                                                 0.0186  0.0023 -0.6129  0.0269  0.0025
                                                -0.0221 -0.0055 -0.0014 -0.0112 -0.0003
                                                -0.0006  0.0019  0.0112
                     ( 25.85%)   0.5084* B 1   s( 14.98%)p 5.64( 84.56%)d 0.03(  0.46%)
                                                 0.0010 -0.3861 -0.0273  0.0033  0.8500
                                                -0.0031 -0.0031 -0.2436  0.0318  0.0004
                                                -0.2506  0.0012  0.0014  0.0206  0.0293
                                                -0.0097 -0.0315  0.0468
96. (1.88341) 3C ( 1) B 2 - B 1 - C 1
                     ( 25.47%)   0.5047* B 2   s(  0.13%)p99.99( 99.68%)d 1.41(  0.18%)
                                                 0.0002 -0.0349 -0.0094 -0.0020  0.0748
                                                 0.0009  0.0022 -0.0617  0.0117 -0.0039
                                                 0.9935  0.0106  0.0117 -0.0070  0.0372
                                                -0.0147  0.0021 -0.0138
                     ( 28.27%)   0.5317* B 1   s(  0.05%)p99.99( 99.33%)d12.25(  0.62%)
                                                 0.0002 -0.0216 -0.0058 -0.0019  0.2507
                                                 0.0018 -0.0000 -0.0799 -0.0117  0.0009
                                                 0.9610  0.0204  0.0023  0.0028 -0.0690
                                                -0.0163 -0.0219  0.0255
                     ( 46.26%)   0.6801* C 1   s(  0.08%)p99.99( 99.87%)d 0.62(  0.05%)
                                                -0.0002 -0.0278  0.0056 -0.0008  0.2214
                                                 0.0094 -0.0013 -0.1537 -0.0029  0.0008
                                                 0.9622  0.0101 -0.0052  0.0064 -0.0064
                                                 0.0190  0.0035  0.0065
```

Supplementary References:

1. Sheldrick, G. M. SHELXT - Integrated space-group and crystal-structure determination. *Acta Cryst.* **A71**, 3-8 (2015).

2. Sheldrick, G. M. Crystal Structure Refinement with SHELXL. *Acta Cryst.* **C71**, 3–8 (2015).

3. Gaussian 09, Revision E.01, Frisch, M. J.; Trucks, G. W.; Schlegel, H. B.; Scuseria, G. E.; Robb, M. A.; Cheeseman, J. R.; Scalmani, G.; Barone, V.; Petersson, G. A.; Nakatsuji, H.; Li, X.; Caricato, M.; Marenich, A. V.; Bloino, J.; Janesko, B. G.; Gomperts, R.; Mennucci, B.; Hratchian, H. P.; Ortiz, J. V.; Izmaylov, A. F.; Sonnenberg, J. L.; Williams-Young, D.; Ding, F.; Lipparini, F.; Egidi, F.; Goings, J.; Peng, B.; Petrone, A.; Henderson, T.; Ranasinghe, D.; Zakrzewski, V. G.; Gao, J.; Rega, N.; Zheng, G.; Liang, W.; Hada, M.; Ehara, M.; Toyota, K.; Fukuda, R.; Hasegawa, J.; Ishida, M.; Nakajima, T.; Honda, Y.; Kitao, O.; Nakai, H.; Vreven, T.; Throssell, K.; Montgomery, J. A., Jr.; Peralta, J. E.; Ogliaro, F.; Bearpark, M. J.; Heyd, J. J.; Brothers, E. N.; Kudin, K. N.; Staroverov, V. N.; Keith, T. A.; Kobayashi, R.; Normand, J.; Raghavachari, K.; Rendell, A. P.; Burant, J. C.; Iyengar, S. S.; Tomasi, J.; Cossi, M.; Millam, J. M.; Klene, M.; Adamo, C.; Cammi, R.; Ochterski, J. W.; Martin, R. L.; Morokuma, K.; Farkas, O.; Foresman, J. B.; Fox, D. J. Gaussian, Inc., Wallingford CT, **2010**.

4. Rincon, L., Almeida, R., Alvarellos, J. E., Garcia-Aldea, D., Hasmy, A., Gonzalez, C. The σ delocalization in planar boron clusters. *Dalton Trans.* 3328-3333 (2009).
